# ADHD and Adolescent Athletes

**DOI:** 10.3389/fpubh.2014.00046

**Published:** 2014-06-17

**Authors:** Ahsan Nazeer, Miriam Mansour, Kathleen A. Gross

**Affiliations:** ^1^Child and Adolescent Psychiatry, Department of Psychiatry, Western Michigan University Homer Stryker M.D. School of Medicine, Kalamazoo, MI, USA; ^2^Department of Psychiatry, Western Michigan University Homer Stryker M.D. School of Medicine, Kalamazoo, MI, USA; ^3^Clinical Research, Department of Psychiatry, Western Michigan University Homer Stryker M.D. School of Medicine, Kalamazoo, MI, USA

**Keywords:** attention-deficit hyperactivity disorder, adolescents, athletes, sports, stimulants, pharmacotherapies

## Abstract

Attention-deficit hyperactivity disorder (ADHD) is a common neurodevelopmental disorder that affects the child and adolescent population. It is characterized by impairment in attention/concentration, hyperactivity, and impulsivity, all of which can impact performance of athletes. ADHD treatment within the athletic population is a unique challenge. The research in this field has been relatively limited. The National Collegiate Athletic Association and International Olympic Committee both regulate the use of psychostimulants for treatment of ADHD due to their performance-enhancing effects. In this article, authors have discussed the screening methods, pharmacological treatment, side effects, and behavioral approaches for the treatment of ADHD in adolescent athletes.

## Introduction

Attention-deficit hyperactivity disorder (ADHD) is a neurodevelopmental disorder that affects about 6–7% of the child and adolescent population (1). Reports suggest that the prevalence of this disorder is higher in athletes at both the collegiate and professional levels. Overall, ADHD is more prevalent in boys (13.2%) than in girls (5.6%), which may explain the above-mentioned discrepancy (2, 3). Literature is limited on how ADHD affects adolescents during sports-related activities. As a result, there is a paucity of literature on the treatment and management of athletes dealing with this disorder. Due to increasing awareness, researchers have started looking closely at the deleterious effects of ADHD in adolescent and adult athletes.

## Search Strategies

For this article, we searched the following databases for the period of January 1985, to December 2013: Medline, PsychINFO, Ovid, PubMed, and Cochrane Library. We used the following search terms: “ADHD,” “adolescents,” “athletes,” and “sports.” Key articles were selected on the basis of topic covered, and emphasis was placed on articles published after 2000. Reference sections of identified publications were also searched for relevant information. The current article is supplemented with the authors’ clinical knowledge, and the references list was modified on the basis of suggestions from peer reviewers.

## Attention-Deficit Hyperactivity Disorder

Descriptions of ADHD in the literature go back to the beginning of the twentieth century. This disorder was further conceptualized and refined at the time that the *Diagnostic and Statistical Manual of Mental Disorders*-*III* (DSM-III) was introduced. ADHD is a chronic disorder that affects multiple areas of life including education, work, family, and social functioning. If left untreated, ADHD can have long-lasting detrimental effects on the individual’s development and psychosocial functioning.

### Signs and symptoms

The *Diagnostic and Statistical Manual of Mental Disorders-IV-TR* identifies inattention, hyperactivity, and impulsivity as the three cardinal symptoms of this disorder (4). DSM-IV-TR also requires onset of these symptoms to be before 7 years of age. Inattentive symptoms include paying poor attention to detail, difficulty sustaining attention, poor follow-through on instructions, poor organization, avoiding homework, losing things, being distractible, and being forgetful. Hyperactivity symptoms include difficulty sitting still, inappropriately leaving one’s seat, fidgeting, running, climbing, being unusually active, and talking excessively. Impulsive behaviors usually present as an individual’s inability to wait for his turn in activities or blurting out answers before being called on in class. Children with a predominance of hyperactive and impulsive behaviors tend to present to clinical care earlier than the ones dealing primarily with inattention (5). Literature has also shown that a significant majority of children continue to deal with this disorder in their late adolescent years and that they do not “outgrow” ADHD (6). This disorder also is associated with significant comorbidity, including mood, anxiety, and oppositional disorders, which further complicates the presentation and treatment (7).

The Diagnostic and Statistical Manual of Mental Disorders-5 continues to include the inattention group criteria as well as the hyperactivity and impulsivity criteria. According to the DSM-5, ADHD symptoms must be present prior to the age of 12 years, in contrast to the age of 7 years as required in the DSM-IV. DSM-5 does not exclude children with autism spectrum disorder (8). ADHD symptoms must not occur during the course of other mental disorders such as schizophrenia, bipolar disorder, anxiety disorder, dissociative identity disorder, personality disorder, and substance intoxication or withdrawal. Such changes to the DSM-5 ensure that children with ADHD continue to receive care throughout their lives as needed.

### Diagnosis of ADHD

In the majority of cases, a thorough clinical interview is sufficient to clearly establish this diagnosis. DSM-5 requires certain criteria to be met before the diagnosis is finalized, including *onset* of the symptoms before 12 years of age, presence of symptoms in more than one setting, and psychosocial dysfunction (8).

Certain tests can be reviewed to further clarify the diagnosis, including the Continuous Performance Test as well as the Connors and the Vanderbilt parent and teacher rating scales. It is important to remember that none of these tests are diagnostic, but they can be used as supporting evidence, especially when the clinical history is equivocal and insufficient by itself to make the diagnosis. On occasion, thorough neuropsychological testing is also warranted. A complete physical examination and laboratory testing are necessary to rule out any medical problems that may be mimicking ADHD symptoms (9).

### Psychosocial development of ADHD adolescents

The adolescent years are marked by rapid psychosocial development with an emphasis on successfully forming an individual identity. During this stage, adolescents face numerous challenges and tend to focus mostly on themselves and their present circumstances. They struggle to improve their self-esteem, which is largely dependent upon their physical attractiveness to others and the role they play in their peer group (10). Adolescents who are successful in this stage grow to be confident and self-assured and go on to find a role in life in which they feel comfortable. Friends and peers tend to play a significant role during this phase of life, and learning to be a part of the team is an important developmental task (11).

Some children with ADHD struggle with these challenges of normal development. Their abilities to deal with the world are somewhat limited in comparison to their non-ADHD peers (12). They grow up having to deal with inattention, disorganization, and limited ability to follow through on instructions. These deficiencies are more pronounced if the ADHD remains undiagnosed and, as a result, untreated. Children with ADHD tend to get more negative feedback from their parents and teachers (13, 14). This in turn affects their self-esteem and negatively impacts their drive to achieve certain goals in life (15). They often feel criticized, yet know that they have limited control over their symptoms (16, 17). Research also has suggested that children with ADHD who feel less able to control their behaviors tend to experience more persistence of these behaviors (18–20).

### ADHD adolescents and sports

Participation in sports is an important part of school and college life. This participation requires individuals to be attentive, organized, and calm and also to have the ability to wait for their turn. These abilities are naturally lacking in children with attention-deficit ADHD. Adolescents with ADHD also tend to struggle with impaired motor coordination, anticipation, and planning (21).

Despite these challenges, adolescents are encouraged to participate in sports, as it is widely accepted that this participation improves on-task behaviors, self-confidence, and social status within the peer group. In one study, authors examined the effects of outdoor activities on ADHD symptoms and concluded that activities in “green” outdoor spaces appear to improve ADHD symptoms the most (22). In another study, a regular exercise program for 5 out of 7 days over 6 weeks significantly improved ADHD-related behaviors in young children and adolescents (23). Other authors have found improved academic performance and overall attitude in children with a regular schedule of physical activity (24). A more recent study examining parental perceptions of the effects of regular physical activity on ADHD showed a positive impact on symptoms, with a greater benefit for symptoms of inattention and hyperactivity compared to symptoms of impulsivity (25).

Adolescents with a hyperactivity disorder report being shy and lonely and are unsure about themselves in a variety of different situations. Social skills training is one of the types of therapy that is used in the treatment of ADHD. Basic techniques in this type of therapy include role playing, modeling, and coaching (26). As with social skills training, participation in sports improves social deficits and also elevates mood and motivation level (27). Adolescents learn social cues and how to trust peers, adhere to rules, and effectively participate in structured activities. This also leads to improved relationships with peers which ultimately improves self-esteem. Participation in sports also affects the neurochemical signals in the brain. Regular exercise increases levels of dopamine and norepinephrine, two vital neurotransmitters that are deficient in individuals with ADHD (28). Elevated levels improve the ability to stay on-task and reduce impulsive behaviors. Regular exercise and other structured activities lead to improved communication between neurons and further neurogenesis (29). Brain-derived neurotrophic factor (BDNF) is the most abundant neurotrophin in the brain (30). BDNF promotes normal brain development, enhances learning and improves neuronal plasticity (31). Reductions in hippocampal BDNF levels are known to impair memory and learning in laboratory animals (32). Exercise is shown to markedly increase levels of BDNF expression in ADHD rat models (33, 34). Exercise has shown improvements in functional, regional, and bio-marker deficits and in hypothalamic pituitary adrenal disruptions (35).

### Management of ADHD

Treatment of ADHD depends upon multiple factors, including the extent of dysfunction in the home, school, or workplace. The age and gender of the individual and the severity of the symptoms also play major roles in the treatment decision-making process. Both medication- and non-medication-related treatments are available, with multiple studies suggesting superior results with medication management. In reality, most adolescents with ADHD require a combination of medication management, academic support with individualized education or 504 plans, and behavioral therapies. Parent training and parent-child interaction therapy are other associated therapies that are available (36).

#### Stimulant medications for the treatment of ADHD

Stimulant medications are usually the first line of treatment for ADHD and are relatively safe (37). These medications have proven efficacy in treating the core symptoms of ADHD, including inattentive, hyperactive, and impulsive behaviors, and numerous studies over the last three decades have validated the safety and effectiveness of these medications (38–43).

Broadly, these medications can be divided into methylphenidate and amphetamine compounds. These compounds enhance activity in both the dopaminergic and noradrenergic systems (44) and thereby improve attention and concentration (45). By affecting dopamine, these medications also have the tendency to increase the heart rate and blood pressure, which is a consideration when prescribing these medications to athletes with ADHD (46). Other noted side effects include abdominal pain, headache, anorexia, sleep impairment, growth problems, weight loss, jitteriness, and constipation (47).

Stimulants medications have profound effects on individuals with and without ADHD and as a result, can be abused in a variety of settings. In non-ADHD adolescents, stimulants improve on-task behaviors, improve concentration, increase the ability to learn more information in limited time, minimize fatigue, and improve the performance level (48–50).

Table 1 is a simplified list of the available stimulant medications and the groups to which they belong.

**Table 1:** **Commonly used stimulant medications by drug class**.

Class	Brand names
Amphetamine	Adderall, Adderall XR, Vyvanse
Methylphenidate	Ritalin, Ritalin LA, Ritalin SR, Concerta, Focalin, Focalin XR, Metadate CD, Methylin
Dextroamphetamine	Dexedrine, Dextrostat

Other medications sometimes used to treat ADHD include atomoxetine, guanfacine, clonidine, bupropion, and the tricyclic antidepressants. These belong to the category of non-stimulant drugs and can be a good alternative in case of concerning side effects from stimulant medications (51).

#### Stimulant medications and effects on athletes

As mentioned above, ADHD causes numerous limitations in the performance ability of adolescent athletes. Limited research is available on the role and effects of stimulant medications in this unique population, but certain reports have suggested that the side effect profile is somewhat different in this population in comparison to non-athletic ADHD adolescents. In one survey, athletes reported lack of creativity, lack of spontaneity, palpitations, sweating, and irritability (52). Overall, it has been noticed that the treated ADHD adolescent athletes have better participation and outcome in sports as compared to their non-treated peers. Stimulants also help them achieve the level of concentration that is necessary for adequate class participation (53).

As stimulants improve concentration and boost performance, some professional athletes in competitive sports consider them beneficial and sometimes use them to gain an advantage over others (54). There is a varied literature about the effects of stimulants on non-ADHD athletes’ sports performance. Research has suggested that these medications do not specifically improve athletic performance but instead improve the *perception* that one is doing well (55). Other studies that have reviewed the effects of these medications on sports have noted improved performance through increased attention to task, improved balance, and enhanced acceleration (56, 57). In a study of college students, amphetamines improved measures of acceleration, although speed and power were not consistently increased (58). Studies have also shown that athletes taking stimulants can exercise at higher core temperatures and heart rates without a perception of increased effort or stress (59, 60). One downside of using stimulants is therefore a potentially increased risk of developing heat-related illness during exercise. On the other hand, the untreated condition of ADHD may offer certain advantages also; for example, impulsivity may translate to spontaneity and quick decision-making.

It is because of the sense of euphoria, perception of slight improvement in certain aspects of performance, and improved concentration that these medications are considered to be *performance enhancing* and are strictly regulated by the National Collegiate Athletic Association (NCAA) and International Olympic Committee (IOC). The NCAA updated their drug testing medical exception procedures in 2008 and now requires more stringent documentation for ADHD diagnosed athletes. It allows for stimulant use by athletes with adequate documentation of ADHD diagnosis and evidence of continued follow up (53, 61). The drug may be present at the time of competition.

In 1992, the IOC introduced the therapeutic use exemption (TUE). Stimulant use by Olympic athletes for ADHD and narcolepsy has been approved recently under the TUE. Stringent rules apply and approval by two independent consultants is required.

Athletes on continued stimulant use must obtain a TUE from the World Anti-Doping Agency (WADA) prior to competing internationally (62). Athletes maintained on continued therapy must undergo annual review. In the case of a well-documented, long-standing diagnosis, a TUE can be granted for up to 4 years; however, yearly reviews by an experienced clinician are still recommended (63).

The true prevalence of ADHD in athletes is not known, as among the athletes who have this condition, few are open about it. Despite the risks, a few athletes have come forward and discussed their condition openly: Michael Phelps, the multiple Olympic gold medalist, was diagnosed with ADHD at 9 years of age; Terry Bradshaw is a pro football Hall-of-Famer who quarterbacked the Pittsburgh Steelers to four Super Bowl victories in the 1970s; Pete Rose became a World Series MVP in 1975; Cammi Granato scored more goals than any other player in the history of U.S. women’s hockey and led her team to a gold medal at the 1998 Winter Olympics in Nagano, Japan. These are just a few examples of the many professional athletes who have excelled despite their disabilities related to ADHD (64).

#### Stimulant medications and cardiac effects

As noted above, stimulant medications have the tendency to increase heart rate and blood pressure (65). Exercise itself has varied effects on the cardiovascular system. During exercise, there is an increased demand for oxygen by other parts of the body, including the brain and muscles. This leads to an increased workload on the heart and a resulting increase in heart rate to meet the demands of the body.

During the last few years, concerns have been raised about the effects of stimulant medications on the cardiovascular system. In 2005, Health Canada suspended the sale of Adderall XR following reports of sudden deaths in children taking this medication. The U.S. Food and Drug Administration (FDA) later issued a warning about the use of these medications in children with pre-existing heart disease. The effects of stimulants on the heart and their association with sudden cardiac death are still being debated, but in 2008, the American Heart Association (AHA) published monitoring guidelines that are currently being followed by most physicians (66).

For varied reasons, the use of stimulant medications in athletes has been an area of concern. It is known that ADHD diagnosed athletes can benefit from these medications, but because of the above-mentioned concerns, a thorough cardiac evaluation needs to be done before the start of medications and before participation in sports (67, 68). Table 2 is a template for an adequate cardiovascular screening before the start of stimulant medications.

**Table 2:** **Cardiovascular screening**.

**PERSONAL HISTORY**
History of chest pain, palpitations, heart murmur, and hypertension
History of structural cardiac defects
History of long and short QT syndrome, right ventricular cardiomyopathy, Brugada syndrome, and Wolff–Parkinson–White syndrome
History of cardiac surgery during infancy
Respiratory symptoms including shortness of breath
Syncope and dizziness
Kawasaki’s disease (fever lasting more than 5 days, lymphadenopathy in neck area, redness of eyes, red lips, and red palms)
Rheumatic fever
**FAMILY HISTORY**
Hypertrophic cardiomyopathy
Premature cardiac death
Marfan syndrome
**CARDIOVASCULAR EXAM**
Heart rate, blood pressure
Palpation of redial and femoral pulses
Cardiac auscultation for systolic ejection murmur, decrescendo diastolic murmur of aortic valve insufficiency, and holosystolic murmur of mitral valve insufficiency
Evaluation for Marfan’s syndrome – consider EKG, slit lamp eye examination, and echocardiography

#### Non-stimulant medications for the treatment of ADHD (69)

##### Atomoxetine (Strattera)

Atomoxetine is a non-stimulant medication that is approved by the FDA for the treatment of ADHD in children. It can be an alternative medication in competitive sports where stimulants are completely banned despite the clinical necessity. Strattera’s side effect profile does not increase the chance of cardiac problems. On the other hand, in 2005, the FDA issued a boxed warning for this drug because of reports of increased suicidality in individuals taking this medication (70). Atomoxetine has also been associated with elevated serum aminotransferase levels (>20 times of normal limit) and acute liver injury in a small percentage (<0.5%) of individuals. The onset of injury was between 3 and 12 weeks of the start of medication. Although most cases were self-limited, a few progressed to acute hepatic failure, requiring liver transplantation (71, 72).

##### Alpha-2 agonists

Clonidine and guanfacine are two other alternatives to stimulants for the treatment of ADHD. Clonidine is the more sedating of the two, but both can cause this undesirable side effect for athletes. Cardiovascular parameters including blood pressure, heart rate, and EKG need to be evaluated before the start of these medications (73). These medications are also available in extended-release (ER) preparations under the brand names Intuniv (long-acting guanfacine) and Kapvay (long-acting clonidine) (74, 75).

#### Behavioral treatment strategies for ADHD athletes

For one reason or an other, neither stimulant nor non-stimulant medications may be the initial choice for adolescent athletes. This is especially true for adolescents with aspirations to compete on the national or international level. In these individuals, implementation of a dedicated behavioral program can help to improve mental capabilities and to overcome ADHD symptoms, namely inattention and impulsivity (76). Problem focused therapies including social skills training, self-control, the stop and think technique, and using positive reinforcements while being consistent and maintaining clear expectations about behaviors are some of the proven strategies (77). The home environment plays a major role in the expression of symptoms of ADHD (78). Homes which provide stability, security, calmness, and structure enable children with ADHD to perform at their best capacity (79).

Mild brain stimulation techniques and neurofeedback (NF) improve brain wave imbalances, facilitating a calmer mental state and therefore better communication throughout the brain (80). NF, which uses signals from the brain’s electrical activity as feedback, has been studied in the last few years for treatment of children with ADHD. In one study, NF was shown to improve the performance of expert rifle shooters (81). Studies have shown that over time, brain stimulation techniques can improve brain development through neuroplasticity. NF is a valid option for treatment, but further studies are needed to determine guidelines for use (80, 82).

## Conclusion

Attention-deficit hyperactivity disorder is a common disorder that affects millions of children all over the world. These children suffer from inattention, hyperactivity, impulsivity, poor self-esteem, and academic and social problems. Because of these symptoms and deficits in motor control, their performance in sports is also impaired (83). Stimulant medications have an extensive literature supporting their safety and effectiveness in the treatment of ADHD. By improving the core symptoms, these medications improve the overall functioning of ADHD adolescents. Recently raised concerns about cardiac problems are debatable (84, 85), but a thorough evaluation by the physician is usually an adequate safeguard against any unwanted side effects. Research is equivocal on whether these medications provide an extra benefit to pro-level athletes. Overall, adequate treatment of ADHD improves the adolescent’s quality of life (86).

## Conflict of Interest Statement

The authors declare that the research was conducted in the absence of any commercial or financial relationships that could be construed as a potential conflict of interest.
